# Physical Activity of ≥7.5 MET-h/Week Is Significantly Associated with a Decreased Risk of Cervical Neoplasia

**DOI:** 10.3390/healthcare8030260

**Published:** 2020-08-10

**Authors:** Ching Wen Chang, Shun-Fa Yang, Christopher J. Gordon, Wen Chun Liao, Shu Fen Niu, Cheng Wei Wang, Hsiu Ting Tsai

**Affiliations:** 1 Department of Obstetrics and Gynecology, Taipei Medical University Hospital, Taipei 11031, Taiwan; ching967@yahoo.com.tw (C.W.C.); fertilitylifetoday@gmail.com (C.W.W.); 2 Institute of Medicine, Chung Shan Medical University, Taichung 40201, Taiwan; ysf@csmu.edu.tw; 3 Department of Medical Research, Chung Shan Medical University Hospital, Taichung 40201, Taiwan; 4 Faculty of Medicine and Health, Susan Wakil School of Nursing and Midwifery, The University of Sydney, Camperdown, NSW 2050, Australia; christopher.gordon@sydney.edu.au; 5 Department of Nursing, School of Nursing, China Medical University; and Consultant, China Medical University Hospital, Taichung 40402, Taiwan; Wcl@mail.cmu.edu.tw; 6 Department of Nursing, Shin Kong Wu Ho Su Memorial Hospital, Taipei 111, Taiwan; niu@tmu.edu.tw; 7 Post-Baccalaureate Program in Nursing, College of Nursing, Taipei Medical University, 250 Wu-Xing Street, Taipei 11031, Taiwan

**Keywords:** cervical neoplasia, leisure-time physical activity, metabolic equivalent of task–hours per week (MET-h/week), human papillomavirus (HPV)

## Abstract

Cervical cancer is one of the most prevalent malignant neoplasms worldwide. We investigated whether leisure-time physical activity is sufficient to decrease the cervical neoplasia risk and provide suggested guidance of metabolic equivalents of task–hours per week (MET-h/week) spent on leisure-time physical activity to prevent cervical neoplasia. Ultimately, 433 participants, including 126 participants with cervical intraepithelial neoplasia I or higher disease (≥CIN 1) and 307 healthy controls, were recruited. All participants completed a standardized questionnaire about leisure-time physical activity engagement (MET-h/week) and a general health questionnaire and had cervical specimens taken to detect human papillomavirus (HPV) infection. CIN 1 staging was identified from the specimens. Participants with physical activity of ≥3.75 MET-h/week had a significantly lower CIN risk compared to those with physical activity of <3.75 MET-h/week (*p* = 0.01). However, among participants with HPV infection or smokers, the minimal requirement of leisure-time physical actively to lessen the CIN risk was ≥7.5 MET-h/week. Lifetime leisure-time physical activity of ≥0.12 MET-h/week–year also significantly decreased the CIN risk, but women with HPV infection needed ≥13.2 MET-h/week–year to protect them from a CIN risk. We concluded that regular leisure-time physical activity of ≥7.5 MET-h/week and sustained lifetime leisure-time physical activity ≥13.2 MET-h/week–year are vital factors for protecting women against cervical neoplasia risk.

## 1. Introduction

Cervical cancer is one of the most prevalent malignant neoplasms and causes of cancer-related deaths worldwide [1]. It was reported that high-risk human papillomavirus (HR-HPV) infection [2,3,4] and tobacco exposure [3,5,6] are the predominant risk factors leading to cervical cancer. However, host immune responses play important roles in protecting against the development of cervical cancer [7,8] and strategies which protect women from cervical cancer risk are strongly needed.

Epidemiological evidence shows that physical activity is associated with decreased incidences of breast, colon, kidney, liver, prostate and endometrial cancers, as well as myelomas and non-Hodgkin lymphomas [9,10,11,12]. While the exact mechanisms are not fully elucidated, physical activity is known to modify immune responses [13,14], which may impact cancer development and progression. In addition, the extent (dose) of physical activity is likely to impact cancer risks, but may have distinct effects on different cancers [9,11,12,15,16]. Therefore, it is important to consider different levels of physical activity when determining the effects on cancer risks.

The metabolic equivalent of task (MET) is a unit of measure of caloric expenditure by the body. It quantifies energy expenditure equivalent to specific physical activities compared to the energy expenditure of an individual at rest (sitting quietly), which is set at 3.5 mL of oxygen per kilogram per minute (1 MET) [10,17]. As such, MET levels can be categorized as strenuous, moderate and mild based on the intensity level during the activity [10,17]. MET hours per week (MET-h/week) is a well-recognized measure of physical activity [18] and physical activity is categorized into five levels: inactive (<3.75 MET-h/week), low volume (3.75–7.49 MET-h/week), moderate volume (7.50~16.49 MET-h/week), high volume (16.50~25.49 MET-h/week) and very high volume (≥25.50 MET-h/week) [18]. Thomson et al. [11] found that physical activity of ≥14.7 MET-h/week combined with adherence to healthy behaviors helped prevent cancers, including breast, colorectal and endometrial cancers [11]. It was reported that nine MET-h/week (moderate volume) of physical activity significantly reduced the incidence of prostate cancer [12], but ≥91.0 MET-h/week (high volume) of physical activity was required to decrease the risk of colon cancer [9]. Friedenreich et al. [15] found that physical activity of >129 MET-h/week/year in women significantly decreased endometrial cancer compared to women with physical activity of ≤82.4 MET-h/week/year. In addition, a light–intensity category (<3 METs) of physical activity of >21.6 h/week/year statistically decreased endometrial cancer compared to a level of physical activity of ≤11.1 h/week/year, but this effect was not found among those with a moderate (3–6 METs) or vigorous (>6 METs) intensity [15]. This suggests that regular lifetime physical activity is a profitable factor to reduce cancer risks and distinct quantities of MET-h/week of physical activity for various cancers are required for cancer prevention [9,11,12,15,16].

We suggest that it is necessary to explore the dosage of MET-h/week of physical activity to protect women from cervical neoplasia; however, only a few studies have evaluated the effects of physical activity on the cervical neoplasia risk [19,20,21]. Szender et al. [19] recruited 128 patients with cervical cancer and 512 participants who were suspected of having but not ultimately diagnosed with a neoplasm to estimate the effect of physical activity on cervical cancer risk. They found that recreational physical activity, but not occupational-related physical activity significantly decreased the cervical cancer risk. However, they did not provide a suggested dose of physical activity for protecting women from cervical cancer [19]. Chih et al. [20] recruited 293 women with a normal Papanicolaou (Pap) smear and 55 women with cervical intraepithelial neoplasia (CIN) screened by Pap smear and found that the volume of physical activity did not significantly differ between these two groups [20]. Lee et al. [21] recruited participants nested within an HPV cohort study to evaluate the association between physical activity and cervical neoplasia. They found that CIN2/3 and cervical cancer risks were negatively correlated with physical activity. Participants with high and medium MET-h/week levels of physical activity exhibited a significant trend of decreasing CIN2/3 and cervical cancer risks compared to participants with lowest tertile MET-h/week levels of physical activity [21]. Nevertheless, none of the three studies [19,20,21] analyzed a stratified effect of physical activity on CIN risk for different smoking exposures and HR-HPV infection statuses, as both smoking exposure and HR-HPV infection are predominant factors inducing cervical neoplasia. The purpose of this study was to investigate whether leisure-time physical activity is sufficient to decrease the risk of cervical neoplasia and provide suggested guidance of MET-h/week levels spent on leisure-time physical activity to prevent cervical neoplasia for the general female population, as well as for specific populations, including women who are non-smokers, are smokers, are exposed to secondhand smoke (SHS), do not have HR-HPV infection and have HR-HPV infection.

## 2. Methods

### 2.1. Participants and Specimen Collection

**Figure 1:**
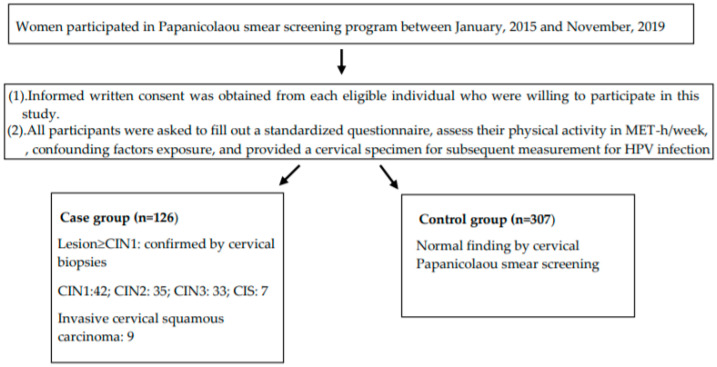
Study flowchart (CIN—cervical intraepithelial neoplasm; CIS—carcinoma in situ).

This was a retrospective hospital-based case-control study. Women who participated in a cervical Pap smear screening program for CIN between 1 January 2015 and 30 November 2019 were invited to participate. Inclusion criteria were women (1) who were aged ≥20 years old, (2) who agreed to participate in the study and (3) who were capable of communicating with the interviewer; (4) who were either with a normal Pap smear or newly diagnosed for the first time as having a lesion ≥CIN1; exclusion criteria were women (1) who were aged <20 years old and (2) who had previously been diagnosed with ≥CIN 1 or any other cancer (3) who with finding of inflammation, preneoplastic and neoplastic lesions of the endocervix with glandular differentiation. All eligible participants who visited the hospital for the cervical Pap smear screening program or who were transferred from a district public health center or clinic for determination of histological results when their Pap smears were abnormal screened in district public center or clinic and were willing to participate in the study were asked to fill out a standardized questionnaire. Their physical activity in MET-h/week and exposure to other confounding factors were assessed by a trained interviewer and they provided a cervical specimen for subsequent measurement for HPV infection to control for the HPV confounding factor. In total, 433 women, including 126 participants who were newly diagnosed for the first time as having a lesion ≥CIN1 and 307 participants with a normal Pap smear, were consecutively recruited for this study. The case group consisted of patients with ≥CIN1 confirmed by a cervical punch biopsy and clinically staged based on the International Federation of Gynecology and Obstetrics Classification at the Department of Obstetrics and Gynecology of Taipei Medical University Hospital (TMUH) (Taipei, Taiwan). The control group consisted of participants with a normal Pap smear. The 307 controls with a normal Pap smear were further verified using colposcopy in a general examination at the outpatient department of Obstetrics and Gynecology at TMUH. The TMUH Institutional Review Board approved the study (ethics no. 201410013) and informed written consent was obtained from each individual. A flowchart of the study is shown in Figure 1.

### 2.2. Sample Size and Statistical Power

A report by Singh et al. [12] stated that a moderate volume of physical activity significantly reduced the incidence of prostate cancer (odds ratio (OR) = 0.47, 95% confidence interval (CI) = 0.22~0.99, *p* = 0.047) [12]. Assuming a 95% CI and *p* = 0.05 and adjusting for confounding factors and stratified analyses, the ratio of cases and controls was 1:2~2.5 and our sample size had at least 85% power to detect a 0.47-fold decreased risk of susceptibility to CIN associated with a moderate volume of leisure-time physical activity.

### 2.3. Collection of Cervical Specimens for the HPV DNA Load

The HPV DNA load was detected by a Hybrid Capture II assay (DIGENE) for high-risk HPV. Details of this analytical method are described elsewhere [5]. Technicians examining specimens for HPV infection in this study were blinded to the findings of Pap smears and cervical biopsies.

### 2.4. Measurement

A previously used baseline questionnaire detailed participant’s demographic characteristics, age, educational level, current and former smoking status, secondhand smoking exposure status and family history of cervical cancer. The validity and reliability of the questionnaire were reported in a previous study [5].

### 2.5. Assessment of Leisure-Time Physical Activity in MET-h/Week

Individuals were asked to complete the questionnaire of regular leisure-time physical activity habits during a typical 7-day period and the number of minutes and how many times on average they completed leisure-time physical activity for more than 15 min for the past 6 months, past 1 year and their lifetime duration [22]. The grades of strenuous effort for physical activity were categorized into mild (minimal effort; heart rate of <120 beats/min; such as yoga, bowling, easy walking), moderate (not exhausting, but some fatigue; heart rate around 120–150 beats/min; such as fast walking, baseball, easy swimming) and strenuous (exhaustion; heart beats rapidly; heart rate above 150 beats/min; such as jogging, soccer, vigorous swimming) physical activity levels. The hours each time and frequencies of these activity levels were multiplied by 3, 5 and 9, respectively. Total weekly leisure-time physical activities were calculated in arbitrary units by summing the products of the separate components using the formula: METS-h/week score = (MET level × hours × times/week). For example, if a participant had 30 min leisure-time of vigorous swimming (strenuous) once per week, 30 min of fast walking (moderate) three times per week and 30 min of easy walking (mild) three times per week, the weekly total MET-h/week score = (9 (strenuous) × 0.5 h/time × 1 time/week) + (5 (moderate) × 0.5 h/time × 3 times/week) + (3 (mild) × 0.5 h/time × 3 times/week) = 4.5 + 7.5 + 4.5 = 16.5 MET-h/week.

### 2.6. Statistical Analyses

Distributions of participant characteristics were examined using a χ^2^ or Fisher’s exact test. An independent *t*-test or a Mann–Whitney *U*-test was used to examine differences between two groups, dependent on normality. The lifetime leisure-time physical activity (MET-h/week–year) was categorized into tertiles according to the distribution among recruited participants. Multivariate logistic regressions were then used to estimate the adjusted ORs (AORs) and 95% CIs of the association between different grades of leisure-time physical activity in MET-h/week and CIN, after adjusting for other covariates. Covariates in the final models were those that were significant or marginally significant in the univariate analysis. A *p*-value of <0.05 was considered significant. We analyzed data using SAS statistical software (version 9.3, SAS Institute, Cary, NC, USA).

## 3. Results

Differences in demographic characteristics and leisure-time physical activity volumes between patients with ≥CIN 1 and healthy controls are shown in Table 1. Of the 126 participants with ≥CIN1 determined by cervical punch biopsies, 42 had CIN1, 35 had CIN2, 33 had CIN3, seven had carcinoma in situ (CIS) and nine had invasive cervical squamous carcinoma. The average leisure-time physical activity (mean ± SE) within the past 1 year was significantly lower (*p* = 0.0001) among patients with ≥CIN 1 (3.67 ± 0.78 MET-h/week) compared to healthy controls (7.7 ± 0.81 MET-h/week). The lifetime leisure-time physical activity (mean ± SE) was also significantly lower (*p* = 0.001) among patients with ≥CIN 1 (25.28 ± 9.19 MET-h/week–year) compared to healthy controls (41.62 ± 6.17 MET-h/week–year) (Table 1).

The AOR and 95% CI of the leisure-time physical activity status and CIN risk among overall participants are shown in Table 2. Participants with leisure-time physical activity of ≥7.5 MET-h/week or 3.75–7.5 MET-h/week had a significantly decreased risk of CIN compared to those with leisure-time physical activity of <3.75 MET-h/week. The AORs were 0.27 (95% CI = 0.14–0.50) and 0.41 (95% CI = 0.19–0.86) for participants with regular leisure-time physical activity of ≥7.5 MET-h/week and those with leisure-time physical activity of 3.75–7.5 MET-h/week within the past 1 year and were 0.27 (95% CI = 0.15–0.49) and 0.42 (95% CI = 0.21–0.85) within the past 6 months, respectively. Lifetime leisure-time physical activity of ≥0.12 MET-h/week–year also significantly decreased the CIN risk. The AORs were 0.18 (95% CI = 0.10–0.32) for participants with lifetime leisure-time physical activity of ≥13.2 MET-h/week–year and 0.23 (95% CI = 0.12–0.44) for participants with lifetime leisure-time physical activity of 0.12–13.2 MET-h/week–year, respectively, compared to those with lifetime leisure-time physical activity of <0.12 MET-h/week–year (Table 2).

The AORs and 95% CIs of the leisure-time physical activity status and CIN risk among participants with and those without HPV infection are shown in Table 3. Among non-HPV participants, there were similar results to those of overall participants (Table 3). However, among participants with HPV infection, the minimal leisure-time physical activity requirement was ≥7.5 MET-h/week and total lifetime leisure-time physical activity was ≥13.2 MET-h/week–year to protect women against CIN risks. AORs and 95% CIs were 0.31 (95% CI = 0.13–0.71) and 0.30 (0.13–0.68) for participants with regular leisure-time physical activity of ≥7.5 MET-h/week within the past 1 year and 6 months, respectively, compared to those with leisure-time physical actively of <3.75 MET-h/week. In addition, AORs and 95% CIs were 0.23 (0.08–0.65) for participants with lifetime leisure-time physical activity of ≥13.2 MET-h/week–year compared to those with lifetime leisure-time physical activity of <0.12 MET-h/week–year (Table 3).

AORs and 95% CIs of the leisure-time physical activity status and CIN risk among non-smokers and active smokers or people exposed to secondhand smoke are shown in Table 4. Among non-smokers, there were similar results to those of overall participants. However, among active smokers or people exposed to SHS, the minimal requirement of leisure-time physical activity was ≥7.5 MET-h/week to protect women against a CIN risk. AORs and 95% CIs were 0.32 (95% CI = 0.11–0.90) and 0.35 (0.13–0.92) for participants with regular leisure-time physical activity of ≥7.5 MET-h/week within the past 1 year and 6 months, respectively, compared to those with leisure-time physical actively of <3.75 MET-h/week. Moreover, AORs and 95% CIs were 0.20 (0.07–0.58) for participants with lifetime leisure-time physical activity of ≥13.2 MET-h/week–year and 0.13 (0.04–0.42) for participants with lifetime leisure-time physical activity of 0.12–13.2 MET-h/week–year compared to those with lifetime leisure-time physical activity of <0.12 MET-h/week–year.

## 4. Discussion

To the best of our knowledge, this is the first study to show an association between the level of leisure-time physical activity and CIN risk for a general female population and specific populations, including non-smoking, smoking, HR-HPV non-infected and HR-HPV-infected women. Our results suggest that regular leisure-time physical activity of ≥7.5 MET-h/week is a vital factor that can protect women against risks of cervical neoplasia.

Interactions of cancer cells with immune cells have crucial impacts on the development of cervical neoplasia [7,8]. Circulating monocytes are involved in a host’s defense during infection or carcinogenesis. However, the tumor microenvironment (TME) destroys the functions of recruited monocytes and causes them to differentiate into tumor-associated macrophages (TAMs), which were found to be associated with cervical carcinogenesis and lymphatic metastasis [7]. TAMs are highly plastic and are modulated by signals from the local tissue environment. Immune phenotypes of TAMs can be classified into Type 1 (classically activated or M1 macrophages) and Type 2 (alternatively activated or M2 macrophages). M1 macrophages possibly contribute to protecting against carcinogenesis by mediating cellular toxicity and the phagocytosis of cancer cells. M2 macrophages are associated with promoting tumor growth and metastasis through immunosuppression and production of angiogenic factors, such as vascular endothelial growth factor (VEGF) and angiopoietin [7,23]. M1 macrophages are modulated by Type 1 helper T (Th1) cells. After exposure to Th1 cytokines, such as tumor necrosis factor (TNF)-α and interferon (IFN)-γ, macrophages adopt the M1 macrophage phenotype [7]. The M2 macrophage phenotype is usually triggered by Th2 cytokines, such as interleukin (IL)-4, IL-13, IL-10 and transforming growth factor (TGF)-β [7]. Li et al. [8] found that the mean number of intratumoral M2 TAMs in cervical squamous cell carcinoma tissue samples was significantly higher than in non-tumorous cervical samples and the number of M2 TAMs was significantly positively correlated with invasion patterns [8]. The 5-year disease-free and overall survival rates were significantly higher among cervical cancer patients with a high M1/M2 ratio in tumor tissues compared to cases with a low M1/M2 ratio [24]. In addition, it was reported that elevation of the Th1/Th2 cytokine ratio could increase cellular immunity and prevent the progression of CIN and invasive cervical cancer [25]. Therefore, it was suggested to develop strategies that promote cellular immune responses, including increasing the Th1/Th2 cytokine ratio, preventing monocyte recruitment to the TME and shifting macrophage phenotypes to M1 macrophages, to prevent the progression of cervical cancer [7].

It was reported that a 3-week exercise training protocol may reduce the recruitment of circulating monocytes into a premalignant TME and can contribute to a reduction in TAMs [26]. Some animal studies reported that physical exercise training benefited the shift of macrophage polarization from M2 to M1 and reduced the production of VEGF and vascularization [27]. Regular exercise was also found to increase Th1 cytokine expressions through upregulating toll-like receptor signaling pathways against pathogen invasion [13,28]. In the present study, among overall participants, leisure-time physical activity of ≥3.75 MET-h/week within the past one year or six months significantly decreased the CIN risk compared to those with leisure-time physical activity of <3.75 MET-h/week, after adjusting for other confounding factors. Also, lifetime leisure-time physical activity of ≥0.12 MET-h/week–year significantly decreased the CIN risk compared to those with lifetime leisure-time physical activity of <0.12 MET-h/week–year, after controlling for other confounders. Our results are consistent with those of Lee et al. [21]. In their study, the highest (72 MET-h/week) and medium (38.5–72 MET-h/week) of physical activity revealed a significant trend of decreasing CIN2/3 and cervical cancer risks compared to women with lowest tertile physical activity of <38.5 MET-h/week [21]. More, women with highest physical activity of 72 MET-h/week protected themselves against CIN2/3 and cervical cancer risks compared to women with lowest physical activity of <38.5 MET-h/week [21]. However, our findings were inconsistent with those of Chih et al. [20], who found that the volume of MET-h/week of physical activity did not significantly differ between women with a normal Pap smear and women with CIN screened by Pap smear [20]. We suggest that a statistically insignificant difference of MET-h/week of physical activity between women with a normal Pap smear and CIN, may be attributed to the CIN not being confirmed by a biopsy and the small size of the study by Chih et al. [20]. We speculated that regular leisure-time physical activity of ≥3.75 MET-h/week or lifetime leisure-time physical activity of ≥0.12 MET-h/week–year can prevent migration of monocytes to the cervical precancerous microenvironment, inhibit the differentiation of monocytes into TAMs and increase Th1 cytokine expressions, which preferentially polarizes TAMs to an M1 phenotype with an anticarcinogenic effect to reduce the CIN risk to women [8,24,28], although the mechanism needs further confirmation in the future.

However, among participants with HPV infection, and those who were active smokers or were exposed to SHS, the minimal requirement of leisure-time physical activity was ≥7.5 MET-h/week to protect women from CIN risk. Moreover, ≥13.2 MET-h/week–year of lifetime leisure-time physical activity was needed to protect women with HPV infection from CIN risk. It was found that HR-HPV infection and tobacco exposure were essential risk factors for developing CIN, particularly for HR-HPV infection which predominantly leads to progression to invasive cervical cancer [5,29,30,31]. The Th1 immune response plays an important role in protecting against the E6 and E7 oncoproteins of HPV 16 and 18 infection and failure of E6-specific Th1 immunity is highly associated with the persistence of HPV infection and CIN progression [29,30]. Th1 cytokines, such as IFN-α and IFN-β, are pivotal in interfering with HPV transcription and activating innate and adaptive immunity [30]. HR-HPVs downregulate the IFN-α-mediated immune response, subsequently benefiting HPV DNA’s movement into the host genome and induction of neoplastic progression in the cervix [32]. In addition, cigarette smoke exposure was reported to suppress the host immune response by decreasing numbers of dendritic cells (DCs), macrophages and nature killer (NK) cells [33]. Moreover, heavy smokers who smoke more than 40 pack–years revealed a predominance of a Th2-type immune response, and this immune response was associated with tumor development [34]. Treating high-grade CIN patients with IFN-α2b was found to increase Th1 cytokine expressions, including IFN-γ, TNF-α and IL-2, and resulted in a significant reduction in the HR-HPV DNA load. Nevertheless, all patients with therapeutic failure were smokers and had higher expressions of the Th2 cytokine, IL-4 or the Th3 cytokines, TGF-β2 and TGF-β3. In addition, it was reported that the HPV E2 protein can increase Th2 cytokine IL-10 expression by directly binding to the IL-10 promoter [35]. Increased expression of IL-10 will shift macrophage phenotypes to M2 macrophages in the tumor environment and induce the T cell regulatory phenotype, which inactivates effector T cells, benefits HPV immune escape and facilitates HPV-associated carcinogenesis, consequently inducing cervical carcinoma [35,36]. A moderate amount of physical exercise was reported to increase expressions of Th1 cytokines, including IFN-γ, TNF-α, IFN-α and IFN-β, and promote immune responses against viral invasion [13]. It was reported that moderate exercise or high-intensity interval training significantly decreased IL-10 expression [37,38]. Our results suggested that women with HR-HPV infection or those exposed to a smoking environment require sufficient Th1 cytokines, particularly IFN-α and IFN-β cytokines, to initiate innate and adaptive immunity against HR-HPV DNA being integrated into the host genome for DNA amplification and prevent a smoking-induced Th2-type immune response [34,39]. The decrease in IL-10 expression for altering macrophage phenotypes from M2 to M1 macrophages and reducing T cell regulatory phenotype is also essential for preventing an HPV escape immune response [36,40]. Moreover, increased expressions of Th1 cytokines and decreased expressions of Th2 cytokines can both strengthen the shift of the immune response to Th1 predominance and inhibit the development of cervical neoplasia [13,37,38]. Therefore, participants with HR-HPV infection or exposed to a smoking environment need at least a moderate level of leisure-time physical activity (≥7.5 MET-h/week) to protect them against CIN risks. In addition, HR-HPV can induce persistent infection by amplifying DNA replication after integration into the host genome [39]. We suggest that lifetime leisure-time physical activity of ≥13.2 MET-h/week–year can contribute to both an increased and accumulative effective immune response against HR-HPV-induced carcinogenesis, although this protective mechanism needs further study.

## 5. Conclusions

Regular leisure-time physical activity of ≥7.5 MET-h/week and sustained lifetime leisure-time physical activity ≥13.2 MET-h/week–year are considered vital factors in protecting women against cervical neoplasia risks.

## 6. Limitations

This study evaluated the effect of leisure-time physical activity on decreasing cervical neoplasia risk, and these findings can be one strategy to protect women against cervical cancer risks. However, there are radically different natural histories and cancerous progression and/or regression for different grades of CIN, such as CIN1, CIN2, CIN3, CIS and invasive cervical cancer. In the future, it is suggested to recruit greater numbers of participants with different grades of cervical neoplasia to estimate the effects of leisure-time physical activity on different grades of cervical neoplasm. More, even though we provided stratification analyses for participants infected with the HR-HPV and those uninfected as well as for participants who were active smokers or exposed to secondhand smoke and participants who were non-smokers. It is important to provide suggested guidance of MET-h/week levels spent on leisure-time physical activity to prevent cervical neoplasia in women who were with both HPV infection and active smokers or people exposed to secondhand smoke. However, our present participant numbers limited the stratified analysis based on four groups of people who smoked and had HPV infection, people who smoked, but did not have HPV infection, people who had HPV infection, but never smoked and people who neither smoked nor had HPV infection. Greater numbers of participants for a four-level stratified analysis is recommend in future study.

## Figures and Tables

**Table 1:** Demographic characteristics.

Variable	Healthy Controls	≥CIN 1	*p*-Value
	(*N* = 307) *n* (%)	(*N* = 126) *n* (%)	
Age (years)			
<41	182 (59.3)	64 (50.8)	*p* = 0.01
≥41	125 (40.7)	62 (49.2)	
Educational level			
<College	66 (21.5)	48 (38.1)	*p* = 0.0004
≥College	241 (78.5)	78 (61.9)	
Smoking status			
Non-smoker	225 (73.3)	78 (61.9)	*p* = 0.03
Exposed to secondhand smoke	46 (15.0)	22 (17.5)	
Smoker	36 (11.7)	26 (20.6)	
HPV infection			
No	228 (74.3)	46 (36.5)	*p* < 0.0001
Yes	79 (25.7)	80 (63.5)	
Family history of cervical cancer			
No	305 (99.3)	122 (96.8)	*p* = 0.06
Yes	2 (0.7)	4 (3.2)	
Average MET-h/week within the past one year	Mean ± SE	Mean ± SE	*p* = 0.0001 ^#^
	7.7 ± 0.81	3.67 ± 0.78	
Lifetime leisure-time physical activity (MET-h/week–year)	Mean ± SE	Mean ± SE	*p* = 0.001 ^#^
	41.62 ± 6.17	25.28 ± 9.19	

χ^2^ or Fisher’s exact test, when appropriate. ^#^ A Mann–Whitney *U*-test was used to examine differences between the two groups. CIN—cervical intraepithelial neoplasm; HPV—human papillomavirus; MET—metabolic equivalent of task.

**Table 2:** Adjusted odds ratio (AOR) and 95% confidence interval (CI) of the leisure-time physical activity status and cervical intraepithelial neoplasm (CIN) risk among overall participants (*N* = 433).

Variable	Healthy Controls (*N* = 307) *n* (%)	≥CIN 1	OR (95% CI), *p*-Value	AOR (95% CI), *p*-Value
		(*N* = 126) *n* (%)		
MET-h/week within the past 6 months				
<3.75	140 (45.6)	90 (71.4)	1.00	1.00
3.75–7.5	56 (18.2)	14 (11.1)	0.38 (0.20–0.74), *p* = 0.004	0.42 (0.21–0.85), *p* = 0.01
≥7.5	111 (36.2)	22 (17.5)	0.30 (0.18–0.52), *p* < 0.0001	0.27 (0.15–0.49), *p* < 0.0001
MET-h/week within the past 1 year				
<3.75	161 (52.4)	95 (75.4)	1.00	1.00
3.75–7.5	52 (17.0)	12 (9.5)	0.39 (0.19–0.77), *p* = 0.0066	0.41 (0.19–0.86), *p* = 0.01
≥7.5	94 (30.6)	19 (15.1)	0.34 (0.19–0.59), *p* = 0.0002	0.27 (0.14–0.50), *p* < 0.0001
Lifetime leisure-time physical activity (MET-h/week–year)				
0~<0.12	90 (29.3)	80 (63.5)	1.00	1.00
≥0.12~<13.2	98 (31.9)	21 (16.7)	0.24 (0.13–0.42), *p* < 0.0001	0.23 (0.12–0.44), *p* < 0.0001
≥13.2	119 (38.8)	25 (19.8)	0.23 (0.14–0.40), *p* < 0.0001	0.18 (0.10–0.32), *p* < 0.0001

AOR adjusted for age, education level, smoking status, family history of cervical cancer and human papillomavirus infection. MET—metabolic equivalent of task.

**Table 3:** Adjusted odds ratio (AOR) and 95% confidence interval (CI) of the leisure-time physical activity status and cervical intraepithelial neoplasm (CIN) risk among participants infected with the human papillomavirus (HPV) and those uninfected (*N* = 433).

	**Without HPV Infection (*N* = 274)**			
**Variable**	**Healthy Controls (*N* = 228) *n* (%)**	**≥CIN 1**	**OR (95% CI), *p*-Value**	**AOR (95% CI), *p*-Value**
		**(*N* = 46) *n* (%)**		
MET-h/week within the past 6 months				
<3.75	102 (44.7)	37 (80.4)	1.00	1.00
3.75–7.5	46 (20.2)	2 (4.4)	0.12 (0.02–0.51), *p* = 0.004	0.12 (0.02–0.55), *p* = 0.006
≥7.5	80 (35.1)	7 (15.2)	0.24 (0.10–0.57), *p* = 0.001	0.25 (0.10–0.60), *p* = 0.002
MET-h/week within the past 1 year				
<3.75	119 (52.2)	39 (84.8)	1.00	1.00
3.75–7.5	43 (18.9)	2 (4.3)	0.14 (0.03–0.61), *p* = 0.008	0.14 (0.03–0.64), *p* = 0.01
≥7.5	66 (28.9)	5 (10.9)	0.23 (0.08–0.61), *p* = 0.003	0.23 (0.08–0.63), *p* = 0.004
Lifetime leisure-time physical activity (MET-h/week–year)				
0~<0.12	68 (29.8)	36 (78.3)	1.00	1.00
≥0.12~<13.2	75 (32.9)	4 (8.7)	0.10 (0.03–0.29), *p* < 0.0001	0.12 (0.03–0.45), *p* = 0.001
≥13.2	85 (37.3)	6 (13.0)	0.13 (0.05–0.33), *p* < 0.0001	0.13 (0.04–0.40), *p* = 0.0004
	**With HPV Infection (*N* = 159)**			
Variable	**Healthy Controls (*N* = 79) *n* (%)**	**≥CIN 1**	**OR (95% CI), *p*-Value**	**AOR (95% CI)*, p*-Value**
		**(*N* = 80) *n* (%)**		
MET-h/week within the past 6 months				
<3.75	38 (48.1)	53 (66.3)	1.00	1.00
3.75–7.5	10 (12. 7)	12 (15.0)	0.86 (0.33–2.19), *p* = 0.75	0.94 (0.35–2.48), *p* = 0.90
≥7.5	31 (39.2)	15 (18.7)	0.34 (0.16–0.73), *p* = 0.005	0.30 (0.13–0.68), *p* = 0.003
MET-h/week within the past 1 year				
<3.75	42 (53.2)	56 (70.0)	1.00	1.00
3.75–7.5	9 (11.4)	10 (12.5)	0.83 (0.31–2.23), *p* = 0.71	0.85 (0.31–2.37), *p* = 0.76
≥7.5	28 (35.4)	14 (17.5)	0.37 (0.17–0.79), *p* = 0.01	0.31 (0.13–0.71), *p* = 0.005
Lifetime leisure-time physical activity (MET-h/week–year)				
0~<0.12	22 (27.9)	44 (55.0)	1.00	1.00
≥0.12~<13.2	23 (29.1)	17 (21.3)	0.37 (0.16–0.83), *p* = 0.01	0.68 (0.24–1.92), *p* = 0.46
≥13.2	34 (43.0)	19 (23.7)	0.27 (0.13–0.59), *p* = 0.001	0.23 (0.08–0.65), *p* = 0.005

AOR adjusted for age, education level, smoking status and a family history of cervical cancer. MET—metabolic equivalent of task.

**Table 4:** Adjusted odds ratio (AOR) and 95% confidence interval (CI) of the leisure-time physical activity status and cervical intraepithelial neoplasm (CIN) risk among non-smokers and active smokers or people exposed to secondhand smoke (*N* = 433).

	**Non-Smokers (*N* = 303)**			
**Variable**	**Healthy Controls (*N* = 225) *n* (%)**	**≥CIN 1**	**OR (95% CI), *p*-Value**	**AOR (95% CI), *p*-Value**
		**(*N* = 78) *n* (%)**		
MET-h/week within the past 6 months				
<3.75	97 (43.1)	55 (70.5)	1.00	1.00
3.75–7.5	45 (20.0)	9 (11.5)	0.35 (0.16–0.77), *p* = 0.009	0.42 (0.18–0.99), *p* = 0.04
≥7.5	83 (36.9)	14 (18.0)	0.29 (0.15–0.57), *p* = 0.0003	0.23 (0.11–0.48), *p* < 0.0001
MET-h/week within the past 1 year				
<3.75	115 (51.1)	58 (74.4)	1.00	1.00
3.75–7.5	40 (17.8)	7 (8.9)	0.34 (0.14–0.82), *p* = 0.01	0.36 (0.15–0.86), *p* = 0.02
≥7.5	70 (31.1)	13 (16.7)	0.36 (0.18–0.72), *p* = 0.003	0.32 (0.16–0.65), *p* = 0.001
Lifetime leisure-time physical activity (MET-h/week–year)				
0~<0.12	65 (28.9)	49 (62.8)	1.00	1.00
≥0.12~<13.2	69 (30.7)	15 (19.2)	0.28 (0.14–0.56), *p* = 0.0003	0.31 (0.15–0.65), *p* = 0.001
≥13.2	91 (40.4)	14 (18.0)	0.20 (0.10–0.40), *p* < 0.0001	0.16 (0.07–0.34), *p* < 0.0001
	**Active smokers or people exposed to secondhand smoke (*N* = 130)**			
Variable	**Healthy Controls (*N* = 82) *n* (%)**	**≥** **CIN1**	**OR (95% CI), *p*-Value**	**AOR (95% CI), *p*-Value**
		**(*N* = 48) *n* (%)**		
MET-h/week within the past 6 months				
<3.75	43 (52.4)	35 (72.9)	1.00	1.00
3.75–7.5	11 (13.4)	5 (10.4)	0.55 (0.17–1.75), *p* = 0.31	0.42 (0.12–1.45), *p* = 0.17
≥7.5	28 (34.2)	8 (16.7)	0.35 (0.14–0.86), *p* = 0.02	0.35 (0.13–0.92), *p* = 0.03
MET-h/week within the past 1 year				
<3.75	46 (56.1)	37 (77.1)	1.00	1.00
3.75–7.5	12 (14.6)	5 (10.4)	0.51 (0.16–1.60), *p* = 0.32	0.55 (0.17–1.80), *p* = 0.33
≥7.5	24 (29.3)	6 (12.5)	0.31 (0.11–0.84), *p* = 0.02	0.32 (0.11–0.90), *p* = 0.03
Lifetime leisure-time physical activity (MET-h/week–year)				
0~<0.12	25 (30.5)	31 (64.6)	1.00	1.00
≥0.12~<13.2	29 (35.4)	6 (12.5)	0.16 (0.06–0.46), *p* = 0.0006	0.13 (0.04–0.42), *p* = 0.0007
≥13.2	28 (34.1)	11 (22.9)	0.31 (0.13–0.75), *p* = 0.01	0.20 (0.07–0.58), *p* = 0.002

AOR adjusted for age, education level, human papillomavirus infection and a family history of cervical cancer. MET—metabolic equivalent of task.
